# Research Progress of Dihydroquercetin in the Treatment of Skin Diseases

**DOI:** 10.3390/molecules28196989

**Published:** 2023-10-09

**Authors:** Ziyang Liu, Dengjun Qiu, Tong Yang, Jingxu Su, Chengyuan Liu, Xinyue Su, Anning Li, Pingping Sun, Jianguo Li, Li Yan, Chuanbo Ding, Shuai Zhang

**Affiliations:** 1 College of Traditional Chinese Medicine, Jilin Agriculture Science and Technology College, Jilin 132109, China; 13656471208@163.com (Z.L.); dengjun_qiu0402@163.com (D.Q.); yangtong20020114@163.com (T.Y.); 13089398468@163.com (J.S.); 18473326206@163.com (C.L.); 15063358905@163.com (X.S.); 2 Jilin Aodong Yanbian Pharmaceutical Co., Ltd., Yanbian Korean Autonomous Prefecture, Dunhua 133700, China; jladcpxstgb@163.com (A.L.); jladpfkl@163.com (P.S.); fengye-jg@163.com (J.L.); yl_yanli@126.com (L.Y.); 3 College of Chinese Medicinal Materials, Jilin Agricultural University, Changchun 130118, China

**Keywords:** dihydroquercetin, skin diseases, skin damage, medical dressing

## Abstract

Skin is a barrier to maintaining the stability of the human environment and preventing the invasion of pathogens. When skin tissue is exposed to the external environment, it will inevitably develop defects due to trauma, injury, burns, ulcers, surgery, and chronic diseases. Rapid skin repair is the key to reducing infection, relieving pain, and improving quality of life. Dihydroquercetin is a kind of flavonoid that has a wide range of pharmacological activities and can improve skin repair, skin inflammation, skin cancer, and so on. In this paper, the application of dihydroquercetin in medical dressings and the research progress in the treatment of skin-related diseases are reviewed, so as to provide reference for further developing dihydroquercetin as a drug for the treatment of skin diseases.

## 1. Introduction

The skin is the coat of the human body, which protects important organs from harm. This important body part is often affected by a series of infections caused by fungi, bacteria, viruses, allergies, dust, and so on, resulting in skin diseases. Skin diseases refer to diseases that occur in the human skin, mucous membranes, and skin appendages [1]. There are many kinds of skin diseases, including more than 200 kinds of common skin diseases, and more than 2000 kinds can be named at present. Skin diseases are not only pathological manifestations of the skin itself but are also closely related to the internal body. In recent years, skin diseases have shown a trend of high incidence and a high recurrence rate, which is increasing year by year [2]. Studies have shown that skin cancer, psoriasis, skin acne, and chloasma have the highest incidence and follow-up rate of skin diseases, and the above diseases are currently intractable skin diseases. Western medicine’s treatment of skin diseases mainly includes oral drugs, external drugs, and physical therapy; however, the side effects of long-term application are obvious, and there are many diseases and complications of skin diseases, which brings many difficulties to clinical treatment [3]. Therefore, it is urgent to find safe drugs that can treat skin diseases. 

Dihydroquercetin (TAX) is a unique bioactive flavonoid. TAX is considered a new food with good market prospects in the United States, the United Kingdom, the European Commission, China, and many other countries. It alleviates acute alcoholic liver injury in mice by regulating NF-κB-mediated inflammation and the PI3K/Akt signaling pathway [4,5]. They have a wide range of pharmacological activities due to their degree of hydroxylation, structural class, other substitutes and conjugates, and the degree of polymerization of metal chelating activity. Dihydroquercetin has shown therapeutic effects on inflammation, cancer, microbial infections, and other diseases. Dihydroquercetin was first extracted and isolated from the leaves of *Chamaecyparis obtusa* (*Siebold et Zuccarini*) *Enelicher* by Japanese scholar Fukui. In recent years, dihydroquercetin has also been found in many fruits, such as olive oil, grapes, citrus fruits, onions, and pine [6]. Studies have found that dihydroquercetin has significant effects in the treatment of skin cancer, skin inflammation, psoriasis, delay of aging, acne, etc. In view of the rich research results of dihydroquercetin in the treatment of skin diseases, this paper reviews the effect of dihydroquercetin in the treatment of skin-related diseases. 

## 2. Classification of Skin Diseases

There are thousands of skin diseases, and there are hundreds of the most common ones. Dermatology, like other specialties, has many classifications. Each has a different etiology, pathology, symptoms, and treatment methods. Correct and effective treatment comes from a correct diagnosis. Common skin diseases are shown in Table 1. 

### 2.1. Skin Cancer

Skin cancer (SC) is a global health threat to a considerable number of people. Various carcinogens (photons, chemicals, and free radicals) cause changes in DNA, proteins, and membranes, which eventually lead to SC [36]. The classification of skin cancer includes two different categories: melanoma skin cancer (MSC) and non-melanoma skin cancer (NMsc) [37]. Among them, non-melanoma skin cancer (NMSC) is the most common type, which affects (slowly) the upper layer of skin due to long-term exposure to ultraviolet rays and is divided into basal cell cancer, squamous cell cancer, and Merkel cell cancer. Other risk factors may cause skin cancer, such as tanning beds, chemical exposure, genetic composition, and immunosuppression. It is very important to avoid skin burns based on ultraviolet radiation and the possible chance of non-melanoma SC. Epidermis is the origin of basal cell carcinoma and squamous cell carcinoma, accounting for 70% and 25%, respectively [38]. From the patient’s point of view, chemotherapy is considered to be the most acceptable form of treatment. However, the major negative effects of chemotherapy, such as severe toxicity and drug resistance, pose a severe challenge to the treatment [37]. 

### 2.2. Atopic Dermatitis

Atopic dermatitis (AD) is a common pruritus and chronic recurrent inflammatory skin disease. The pathophysiology of AD includes skin barrier dysfunction, frequent allergic reactions to allergens, defects in antibacterial immune defense, and genetic susceptibility [39]. Severe itching and skin reactivity are the main characteristics of AD [40], and they are the main burden for patients involved. Therefore, it is very important to understand the underlying mechanism of chronic itching in AD patients, not only for understanding the course of the disease but also for developing new treatment strategies to alleviate this main clinical symptom [41].

### 2.3. Psoriasis

Psoriasis is one of the most common chronic inflammatory diseases [42]. It has become a major health and social burden all over the world. Psoriasis is characterized by the infiltration of inflammatory cells and the abnormal proliferation and differentiation of epidermal keratinocytes, accompanied by increased capillary permeability, blood flow, and blood vessel formation. This series induces the development of psoriasis lesions, which are characterized by obvious red scales and erythema on the skin, accompanied by itching and pain [43], which are caused by pro-inflammatory cytokines released by cells of the innate and adaptive immune systems and can cause long-term lesions [44].

### 2.4. Skin Acne

Acne vulgaris is a disease of sebaceous glands in hair follicles and the eighth most prevalent disease in the world, affecting 9.4% of the global population [45]. Acne is a disease with complex pathophysiology that is caused by immune and inflammatory processes, sebaceous gland hyperplasia, microbial overgrowth, and follicular hyperkeratosis [46]. Acne vulgaris is characterized by acne, inflammatory spots, and subsequent pigmentation [47], which may have a great social and psychological impact because it is easy to see, has a slow response to treatment and a chronic disease course, and has heavy physical consequences [48].

### 2.5. Chloasma

Chloasma is a common acquired hyperpigmentation skin disease that is characterized by light brown or dark brown patches, usually symmetrically distributed, and is most common in the cheeks, forehead, and other photosensitive parts. Its incidence runs in the family and is also influenced by gender, age, and environmental factors. The main reasons are ultraviolet radiation, family history, pregnancy, and the use of exogenous sex hormones. In addition, chloasma can occur in almost all skin types, and the prevalence rate of men and women varies according to race. Because the repeated symptoms last for a long time, it seriously affects people’s quality of life and may also cause mental and psychological problems such as anxiety, irritability, and depression [49].

This section introduces the characteristics and effects of skin cancer, skin inflammation, psoriasis, skin acne, and chloasma. We hope this will help to develop drugs with potential therapeutic mechanisms for skin diseases and deepen our understanding of skin diseases. 

## 3. Skin Dressing

Traditional treatments for skin lesions include the use of protective wound dressings or more invasive methods, such as tissue transplantation (removal from a patient’s donor area or allogenic source). These treatments may be inefficient or infeasible. Because of the affected areas, tissue rejection, pollution, and necrosis risk, it is of great scientific and clinical significance to develop biomaterials for skin regeneration [50]. Some polymers, such as chitosan (CS) and hyaluronic acid, are famous for their bioadhesion and biocompatibility in the pharmaceutical field and have been proven to promote the process of tissue repair; therefore, they are ideal candidates for the treatment of skin lesions [51]. In the treatment of skin wounds, this biomaterial is usually used in the form of a dressing or scaffold. The advanced wound dressing should mainly aim at preventing bacterial infection by fully sealing the wound microenvironment from external pollutants and should be able to control the proliferation of bacteria (or fungi), limit the incidence of infection, and possibly eradicate pathogens [52] to achieve the effect of treating skin diseases. 

Modern wound dressings are becoming more and more advanced, which can provide a suitable environment for the natural healing process or target the transfer of molecules needed for healing. The treatment of epithelial and skin lesions is gradually changing, from the traditional method based on the use of disinfectants and medical equipment to the modern method based on the use of bioactive polymers that can promote wound healing. Studies have found that gels and dressings loaded with drugs in particular have been used to treat mucosal, corneal, and skin lesions [53]. In terms of pain and infection management, encapsulating drugs in wound dressings is a promising alternative to systemic antibiotics and pain management therapy [54]. Therefore, drug compounds can be loaded on the surface of the dressing and released to the dermis of the skin for the treatment of skin diseases. 

## 4. Application of Dihydroquercetin in the Treatment of Skin Diseases

Dihydroquercetin has a wide range of pharmacological activities of flavonoids, including antioxidation, antivirus, and anti-inflammation [55], and has inhibitory effects on inflammation, malignant tumors, microbial infection, and other diseases [56] Based on these favorable activities, dihydroquercetin also plays a certain role in the treatment of skin diseases (Table 2).

### 4.1. Treatment of Skin Cancer

Skin cancer is one of the most common cancer incidence rates in the world, and dihydroquercetin has good activity in the pre-treatment of skin cancer. Studies have shown that dihydroquercetin binds to EGFR and PI3-K at the adenosine triphosphate (ATP) junction and inhibits their protein kinase activity. It can also inhibit UVB-induced EGFR-related Akt protein activation and prevent the downstream signal cascade reaction in JB6P + mouse skin epidermal cells. Local application of dihydroquercetin can inhibit the incidence and volume of tumors in a mouse model, which indicates that dihydroquercetin has a preventive effect on chemical skin cancer induced by UVB by regulating EGFR and PI3K targets [57].

There are many factors that cause cancer and scars, including vaccines, burns, and other injuries. These cancers are called scar cancers and usually occur in skin tissues [58]. Dihydroquercetin’s research on the anticancer activity of skin scar cell carcinoma (SSCC) shows that dihydroquercetin can effectively inhibit the development of SSCC by inducing apoptosis and blocking the cell cycle. Dihydroquercetin can also inhibit the invasion of SSCC by down-regulating the expression of MMP-2 and MMP-9 [59]. Topical application of dihydroquercetin to the skin of mice can significantly reduce the incidence of tumors induced by SUV [57]. Local application of dihydroquercetin reduced the average tumor volume of each mouse and significantly reduced the tumor weight at the 30th week. Protein blot analysis of mouse skin showed that the expression levels of EGFR, Akt, and COX-2 proteins and their phosphorylation were significantly decreased in the dihydroquercetin treatment group. Dihydroquercetin also effectively inhibited the production of PGE2 in mouse skin. These results indicate that dihydroquercetin has a strong preventive effect on skin carcinogenesis induced by SUV in mice.

### 4.2. Treatment of Psoriasis

Psoriasis is a skin disease with an autoimmune tendency. It is reported that imiquimod can induce excessive proliferation and abnormal apoptosis of epidermal cells in a psoriasis mouse model. The analysis of the enriched KEGG pathway shows that the PPAR-γ pathway is the most involved in the pathogenesis of psoriasis, and dihydroquercetin significantly reduces the increase of p-cPLA2 and PPAR-γ protein levels in keratinocytes and pathological tissues induced by imiquimod, respectively. These results indicate that dihydroquercetin can prevent imiquimod-induced excessive immune activation and keratinocyte proliferation by reducing p-cPLA2 and regulating the PPAR-γ pathway [60]. Dihydroquercetin can effectively inhibit the abnormal proliferation of Hacat cell lines induced by LPS, mainly by regulating the Jak1/stat1 signaling pathway to inhibit ICAM-1 expression in Hacat cells induced by IFNγ [61]. In addition, dihydroquercetin may have an effect on keratinocytes under inflammatory conditions and significantly improve psoriasis induced by IMQ in BALB/c mice. Dihydroquercetin can also regulate Th cells by inhibiting the differentiation of several transcription factors (such as T-bet, GATA-3, and RORγt). In addition, it can also inhibit the activation of Notch1 and Jak2/Stat3 signaling pathways, thus alleviating psoriasis [62].

### 4.3. Treatment of Skin Inflammation

Skin inflammation is a general term for skin inflammatory diseases caused by various internal and external infections or non-infectious factors. It is not an independent disease, and its etiology and clinical manifestations are complex and varied, making it difficult to treat clinically. Dihydroquercetin has anti-inflammatory pharmacological effects and can effectively improve skin inflammation [63]. Compared with the aqueous solution, the EL, or Pep-1 peptide mixed EL preparation, the PEP-1-EL preparation loaded with dihydroquercetin nanoparticles has better absorbability. Compared with the control group treated with dihydroquercetin aqueous solution, the skin barrier function of the Pep1-EL group loaded with dihydroquercetin showed a significantly accelerated recovery trend (*p* < 0.05). At the same time, local application of Pep1-EL loaded with dihydroquercetin can also regulate the immune response related to atopic dermatitis [64]. After dihydroquercetin glucoside treatment, the levels of eosinophils and IgE in mice decreased, and the expression levels of cytokines such as IL-4, IL-5, and IL-13 in the dihydroquercetin treatment group decreased, indicating that dihydroquercetin glucoside may be related to the immunoregulation related to specific dermatitis. At the same time, the expression levels of iNOS and COX-2 in the dihydroquercetin glycoside treatment group showed a significant decrease [65]. This shows that dihydroquercetin can effectively treat atopic dermatitis by reducing the production of inflammatory cytokines and skin inflammation. Dihydroquercetin can regulate the expression of the ICAM-1 protein at the transcription level. Among the flavonoids detected, dihydroquercetin is the most effective in inhibiting the ICAM-1 protein induced by interferon γ(IFNγ) and mRNA expression in human keratinocytes [61]. These results indicate that dihydroquercetin has good therapeutic potential in the treatment of skin diseases related to increased cell adhesion and inflammation.

### 4.4. Delay Skin Aging

Dihydroquercetin is a natural inhibitor of CD38, which consumes NAD+, and dihydroquercetin can delay aging by increasing the level of NAD+. In addition, dihydroquercetin can inhibit the aging process by keeping the skin structure intact, reducing the degree of collagen decomposition and skin oxidative stress, protecting collagen and its downstream related proteins from degradation, and further down-regulating the expression of apoptotic proteins, thus achieving the purpose of protecting the skin and delaying skin aging [66].

### 4.5. Treatment of Skin Acne

Batubara [67] et al. evaluated the anti-acne efficacy of flavonoid glycosides isolated from Kempas bark (Koompassia malaccensis) and determined their antibacterial efficacy against Propionibacterium acnes, lipase inhibitory and antioxidant activities, and tyrosinase inhibitory activities. The results showed that all the compounds had no significant antibacterial activity against Propionibacterium acnes, while dihydroquercetin showed good lipase inhibition and antioxidant activity. However, TAX showed 24.12% and 5.18% inhibition of l-tyrosine and L-DOPA inhibition of tyrosinase activity, respectively. It shows that dihydroquercetin has the efficacy of treating skin acne.

### 4.6. Dihydroquercetin Promotes Wound Healing

The cost of wound healing treatment is high, wound recovery is slow, and the uncertainty of human physiological activities poses a serious burden on public health [68]. The process of wound healing is divided into four stages, which are hemostasis, inflammation, proliferation, and remodeling. Zhang et al. [63] found that pretreatment of dihydroquercetin composite nanofiber membrane can prevent inflammation, apoptosis, and oxidative stress signals during the overexpression of the UVA exposure pathway induced by MAPK (p-ERK, p-JNK, and p-P38)/nrf2. Immunofluorescence experiments also show that a dihydroquercetin composite nanofiber membrane can reduce the fluorescence intensity of Caspase-3 and TNFα. The results show that CPD may be a successful healing agent, providing enhanced anti-uva-induced oxidation and skin irritation compensation. Another study by Jinping Zhang et al. [68] found that a dihydroquercetin-loaded composite nanofiber membrane was prepared by electrospinning technology. In vivo analysis, the CPD composite nanofiber membrane promoted angiogenesis by promoting the expression of VEGF and CD31, which was beneficial to skin repair. Regulating autophagy may be helpful to treat skin barrier dysfunction [69]. Studies have shown that p75NTR silence activates autophagy to inhibit proliferation, migration, and extracellular matrix deposition of hypertrophic scars, thus promoting wound healing [70].

### 4.7. Dihydroquercetin Treatment of Skin Burns

The application of preparations based on free flavonoids and their complexes with Fe(II/III) and Cu(II) ions after chemical burns can more effectively regenerate skin and repair hair follicles and sebaceous glands. Compared with the control group, it was observed that dihydroquercetin -Cu(II) and dihydroquercetin -Fe(III) liposome complexes could promote wound healing more effectively [71]. Effect of liposome preparations containing dihydroquercetin protein oligomers and conjugates of dihydroquercetin and carbonyl compounds on skin regeneration after chemical burns The results showed that the preparation containing fluorine-like compound conjugates enhanced the regeneration process and repair of hair follicles and sebaceous glands after chemical burns. It shows that the conjugate of dihydroquercetin and carbonyl compounds can be used to create composite preparations for wound and burn healing [72].

### 4.8. Treatment of Skin Ulcer and Chloasma

Cadmium (Cd) is a highly toxic chemical substance and a major industrial pollutant in developed countries. It is widely distributed in air, food, soil, sediment, and water. SH Moon et al. [73] studied the protective effect of dihydroquercetin on apoptosis of HaCaT keratinocytes induced by Cd. The results showed that the cell death induced by Cd in cells treated with dihydroquercetin (0–100μM) was lower than that in cells treated with Cd alone. The level of reactive oxygen species (ROS) in cadmium/dihydroquercetin-treated cells was also lower than that in CD-treated cells. Dihydroquercetin has a protective effect on Cd-induced apoptosis cells, which is manifested in the changes in caspase 3, −7, and ADP- ribose polymerase activities. In addition, the level of cell cycle-related proteins (such as SP1 and p21) decreased while the level of p53 increased, indicating that dihydroquercetin reduced Cd-induced cytotoxic cell apoptosis by inhibiting apoptosis.

## 5. Dihydroquercetin Loaded Wound Dressing

Dihydroquercetin has abundant pharmacological activities and has the efficacy of treating skin diseases [74,75]. However, due to its low utilization and poor water solubility, the local delivery of dihydroquercetin to the relevant skin layers is obviously limited. Therefore, the lack of an effective delivery system is still a major obstacle to the development of topical dihydroquercetin preparations [76]. Now dihydroquercetin is loaded into wound dressings for the repair of skin lesions (Table 3).

**Figure 1:**
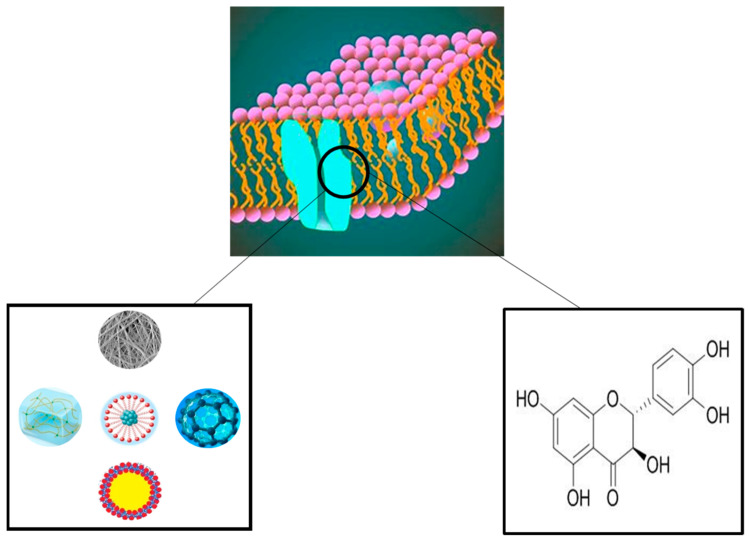
Schematic diagram of a nanofiber membrane loaded with dihydroquercetin.

The porous structure and excellent pore interconnectivity of nanofibers make them an ideal choice for wound dressing and wound healing because they have oxygen permeability, the ability to keep moisture at a required level, an inhibition effect on the invasion of exogenous microorganisms, and the ability to conform to the skin of the wound site and reduce scars [77,78,79]. Some studies have reported nanofiber membranes loaded with dihydroquercetin. As shown in Figure 1.

**Figure 2:**
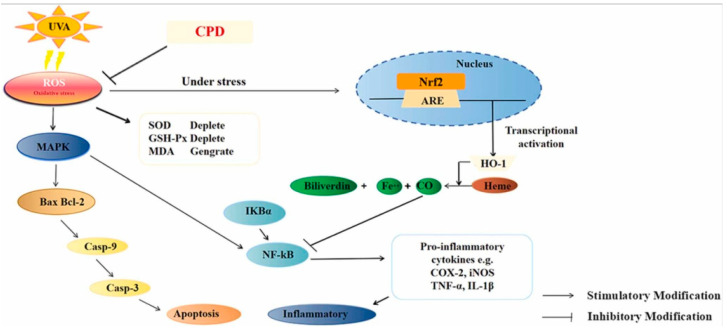
Dihydroquercetin can prevent inflammation, apoptosis, and oxidative stress mechanisms caused by UVA radiation [63].

In order to inhibit the formation of wound scars, Ding et al. mixed TAX and dihydroquercetin protein liposome (TL) with polyvinyl alcohol (PVA) and CS by electrostatic spinning to prepare a nanocomposite membrane [80]. The results show that PVA/CS/TL can increase the expression of CD and VEGF in skin tissue and promote the wound healing of diabetic mice by inhibiting the activation of the κB α(IκBα)/nuclear factor κ B (NF-κB) signaling pathway and related pro-inflammatory factors. Dihydroquercetin composite nanofiber membrane (CPD) was prepared by Zhang et al. [63], and the protective effect of UVA on human skin keratinocytes (HaCaT) was studied. The results show that CPD can prevent the light damage of human skin keratinocytes by preventing oxidative stress, inflammation, and apoptosis induced by the MAPK/Nrf2 signaling pathway induced by UVA radiation. CPD may be an effective defense and treatment formula to prevent UVA-induced skin photodamage (Figure 2). In another study by Zhang et al. [68], CS, PVP, and dihydroquercetin (DHQ) nanofiber films were prepared. The results show that it has antibacterial activity against *Staphylococcus aureus* and *Escherichia coli*. Animal experiments show that wounds treated with the CS-PVP-DHQ nanofiber membrane heal faster. Mohamed A. Hassan et al. [81] prepared a multifunctional polyelectrolyte wound dressing membrane based on Cs and hyaluronic acid (HA) and reinforced by phosphatidylcholine dihydroquercetin (PCDQ). This study showed that CS/HA/PCDQ film significantly improved the full-thickness wound in mice, and the reduction of wound area proved this. In addition, compared with the wound treated with cotton cloth and CS/HA dressing, the histological examination of the wound wrapped with CS/HA/PCDQ shows that the healing speed is obvious compared with the wound treated with cotton cloth and CS/HA dressing, which reveals the efficiency of PCDQ. These findings emphasize that the CS/HA/PCDQ membrane has outstanding potential for wound healing and skin regeneration.

In the above research, different materials were used to prepare nanofiber membranes loaded with dihydroquercetin, which improved the stability of dihydroquercetin. Adding hydrophilic substrates such as CS, HA, and PVA to nanofiber membranes also improved the bioavailability of TAX. From the mechanism of action of TAX-loaded nanofibers on wound healing, it can be seen that TAX-loaded nanofibers mainly play an active role in the inflammatory and proliferative stages of wound healing by regulating the expression of inflammatory factors and vascular growth factors. Therefore, the nanofibers containing TAX can be widely used in the field of skin repair and promote wound healing.

## 6. Conclusions

Various skin diseases have a great impact on our normal lives; therefore, it is imperative to find a drug that can treat skin diseases. As a flavonoid compound, dihydroquercetin has good biological activity and has significant effects on the prevention and treatment of skin diseases such as skin cancer, psoriasis, skin inflammation, and skin aging. We have summarized its effects and mechanisms in the treatment of common skin diseases, providing more possibilities for the utilization of dihydroquercetin.

In daily life, skin injuries often occur. At present, most clinical treatments for skin injuries include antibiotic treatment, cytokines and growth factors, wound debridement, etc. However, there are problems such as drug resistance, difficulty in clinical application, expensive treatment, and insignificant effects. Traditional dressings, such as gauze, may adhere to new granulation tissue, cause pain and affect tissue integrity when removed, and do not have antibacterial, antioxidant, or other active functions. Dihydroquercetin is added to medical dressings, allowing dihydroquercetin to be applied to skin areas to fully exert its functions. Through potential mechanisms such as dihydroquercetin’s antioxidant and anti-inflammatory effects, it promotes wound healing, inhibits scar formation, and regulates inflammatory factors and angiogenesis. This may provide additional opportunities for the treatment of skin diseases and lay the foundation for the development of future wound dressings.

## Figures and Tables

**Table 1:** Common classification of skin diseases.

Serial Number	Skin Diseases	Represents Disease	References
1	Skin cancer	Basal cell carcinoma of the skin, squamous cell carcinoma, malignant melanoma, nodule	[7,8,9]
2	Inflammatory dermatosis	Psoriasis, atopic dermatitis, autoimmune vesicular disease, and alopecia areata	[10,11]
3	Erythematopapular scaling dermatosis	Psoriasis, subcorneal pustular skin disease, pityriasis rosea, seborrheic dermatitis, and nummular dermatitis	[12]
4	Skin and accessory organ diseases	Acne, rosacea, skin ulcers, etc	[13,14,15]
5	Pigment disorder is a skin disease	Chloasma, vitiligo, pigmented nevus, freckle nevus, etc.	[16,17,18,19]
6	Viral skin diseases	Herpes simplex, herpes zoster, warts, molluscum contagiosum, hand, foot, and mouth disease	[20]
7	Bacterial dermatosis	Impetigo, folliculitis, erysipelas, and leprosy	[21,22]
8	Fungal dermatosis	Tinea corporis, tinea pedis, onychomycosis, pityriasis versicolor, malassezia folliculitis, etc	[23]
9	sexually transmitted disease	Syphilis, gonorrhea, and condyloma acuminatum	[24,25]
10	allergic skin diseases	Contact dermatitis, eczema, urticaria, drug reaction	[26,27]
11	Neurofunctional dermatosis	Prurigo nodosa, neurodermatitis, and parasitic paranoia	[28,29]
12	Connective tissue diseases	Lupus erythematosus, scleroderma, and dermatomyositis	[30]
13	Bullous skin diseases	Pemphigus, bullous pemphigoid, cicatricial pemphigoid, herpetic dermatitis, linear IgA bullous dermatosis, acquired epidermolysis bullosa, pemphigoid pregnancy	[31]
14	Genodermatoses	Ichthyosis vulgaris, keratosis folliculi, albinism, hirsutism, epidermolysis bullosa, tinea versicolor	[32,33]
15	Nutrition and disordered metabolic dermatosis	Vitamin deficiency	[34,35]

**Table 2:** Mechanism of bioactivity of dihydroquercetin.

Biological Activity	Experimental Model	Mechanism
Anti-inflammatory	C57BL/6 mice	Regulating the TLR4/NF-κB axis to prevent inflammation and apoptosis.
	RAW 264.7 cells	Regulates the expression of iNOS, VEGF, COX-2, and TNF-α and affects the MAPK signaling pathway.
	SD rat	Inhibition of microglial pyroptosis via the PI3K/Akt signaling pathway
	DMM rats and chondrocytes	Activating the Nrf2 pathway inhibits inflammation, alleviates apoptosis, and reduces ECM degradation to reshape the articular cartilage microenvironment.
	BV2 cell line	Upregulates pAMPK levels and activates the Nrf2/HO-1 signaling pathway
Antibacterial	*E. coli* and *Staphylococcus aureus*	Destroy the integrity of bacterial cell walls and membranes, inhibit bacterial biofilm formation, cause stress, and lead to increased superoxide dismutase and alkaline phosphatase activities in bacteria
Antioxidants	Westal rat	Reduce oxidative stress and reduce pro-inflammatory cytokine levels
	glutamatergic neurons	Inhibition of basal and OGD-induced mitochondrial ROS production in GABAergic neurons.
Antiviral	HAV stock: CF 53	Reduce the infectivity and antigenicity of HAV

**Table 3:** Dihydroquercetin dressing promotes skin damage repair function.

Dressing Type	Experimental Model	Mechanism
Nanofiber membrane	Diabetic mice	It Inhibits the activation of κBα (IκBα)/nuclear factor κB (NF-κB) signaling pathway and increases the expression of CD and VEGF in skin tissue
	Human skin keratinocytes	Prevent oxidative stress, inflammation, and apoptosis induced by UVA radiation-induced MAPK/Nrf2 signaling pathway
	Mice	The PI3K/AKT/MR signaling pathway is significantly inhibited to activate autophagy and promote skin repair.
	Female Wistar rats	Good antioxidant, antibacterial, and anti-inflammatory activity.

## Data Availability

Data supporting the findings are available by the corresponding authors upon reasonable request.
